# Inhibitory Effects of Palmultang on Inflammatory Mediator Production Related to Suppression of *NF-κB* and *MAPK* Pathways and Induction of *HO-1* Expression in Macrophages

**DOI:** 10.3390/ijms15058443

**Published:** 2014-05-13

**Authors:** You-Chang Oh, Yun Hee Jeong, Won-Kyung Cho, Min-Jung Gu, Jin Yeul Ma

**Affiliations:** Korean Medicine (KM)-Based Herbal Drug Development Group, Korea Institute of Oriental Medicine, 461-24, Jeonmin-dong, Yuseong, Daejeon 305-811, Korea; E-Mails: ulivuli@kiom.re.kr (Y.-C.O.); runxi0333@kiom.re.kr (Y.H.J.); wkcho@kiom.re.kr (W.-K.C.); guminjung@kiom.re.kr (M.-J.G.)

**Keywords:** palmultang, inducible nitric oxide synthase, heme oxygenase-1, nuclear factor-kappaB, mitogen-activated protein kinase

## Abstract

Palmultang (PM) is an herbal decoction that has been used to treat anorexia, anemia, general prostration, and weakness due to chronic illness since medieval times in Korea, China, and Japan. The present study focused on the inhibitory effects of PM on the production of inflammatory factors and on the activation of mechanisms in murine macrophages. PM suppressed the expression of nitric oxide (*NO*), inflammatory cytokines and inflammatory proteins by inhibiting nuclear factor (*NF*)*-κB* and mitogen-activated protein kinase (*MAPK*) signaling pathways and by inducing heme oxygenase (*HO*)*-1* expression. Collectively, our results explain the anti-inflammatory effect and inhibitory mechanism of PM in macrophages stimulated with lipopolysaccharide (LPS).

## 1. Introduction

Palmultang (PM) is a traditional herbal medication that has been used since medieval times in East Asia. Currently, PM is usually prescribed as an herbal medicine for the treatment of various symptoms associated with body weakness. Previous studies demonstrated that PM was an effective treatment for endometriosis [1]. In addition, a recent study revealed that PM has a beneficial effect on reproductive function in female mice [2]. However, the effects of PM on inflammation and inflammatory mechanisms still remain unknown.

Macrophages play a key role in the regulation of inflammatory and immune responses [3,4]. Activation of macrophages is induced by LPS stimulation, and activated macrophages secrete inflammatory factors, such as *NO*, prostaglandin (*PG*)*E_2_* and inflammatory cytokines [5,6]. *NO* and *PGE_2_* are synthesized by inducible nitric oxide synthase (*iNOS*) and cyclooxygenase (*COX*)*-2*, respectively, and the expression of *iNOS* is closely related to the induction of *HO-1. HO-1* is a stress-inducible protein that catalyzes the oxidative degradation of heme; two other heme oxygenase isoforms, *HO-2* and *HO-3*, have also been identified [7]. Enhancing the production of *HO-1* reduces the expression of *iNOS* and the level of free radicals [8].

*NF-κB* plays an important role in the expression of inflammatory genes. When unstimulated, *NF-κB* is present in the cytoplasm attached to *IκBα; NF-κB* is released through degradation of *IκBα* when induced by LPS [9]. Activated *NF-κB* can be transferred from the cytoplasm to the nucleus, where it binds to promoters and induces the expression of various inflammatory genes [10,11]. *MAPK* signaling pathways play an important role in transmitting inflammatory signals [12] and comprise extracellular signal-regulated kinase (*ERK*), *p38*, and c-Jun NH_2_-terminal kinase (*JNK*) pathways. *MAPK*s are activated by phosphorylation and induce activation of the *NF-κB* pathway and expression of the *iNOS* gene.

In the present study, we evaluated the suppressive effect of PM on inflammation induced by LPS in RAW 264.7 macrophages. Further, we researched whether the effects of PM on *NF-κB* and *MAPK* signaling pathways and on induction of *HO-1* explain the anti-inflammatory mechanism of PM.

## 2. Results and Discussion

### 2.1. PM Did not Show Cytotoxicity and Had Inhibitory Activity against NO and Inflammatory Cytokine Production in Macrophages

**Figure 1.**
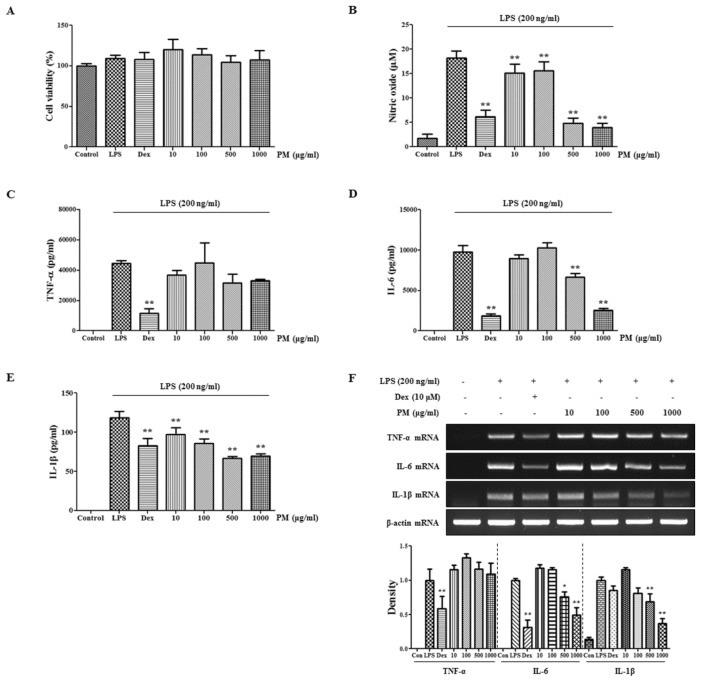
(**A**) The cytotoxicity of PM in RAW 264.7 cells. And the suppressive effect of PM on (**B**) *NO* production and (**C**–**F**) *TNF-α*, *IL-6* and *IL-1β* expression induced by LPS stimulation in macrophages. RAW 264.7 cells were pretreated with PM for 30 min before incubation with LPS for (**A**–**E**) 24 h or (**F**) 6 h. (**A**) Cytotoxicity was determined using CCK; (**B**) The culture supernatant was analyzed for nitrite production; (**C**–**E**) Production of cytokines was measured by ELISA and (**F**) mRNA levels were analyzed by RT-PCR. RNA values were quantitated using the i-MAX™ Gel Image Analysis System (Core Bio, Seoul, Korea). As a control, cells were incubated with vehicle alone. * *p* < 0.01 and ** *p* < 0.001 were calculated via comparisons with the LPS-stimulation value.

In the present study, we demonstrated anti-inflammatory activity of PM in murine macrophages stimulated with LPS. First, we investigated the cytotoxicity of PM in RAW 264.7 macrophages at concentrations of 10–1000 μg/mL. As shown in Figure 1A, PM did not show cytotoxicity at concentrations up to 1000 μg/mL, indicating that it is not toxic to macrophages. Based on this result, we did experiments using up to 1000 μg/mL concentrations of PM.

The overproduction of *NO* is associated with various inflammatory diseases [13,14], so we preferentially investigated the inhibitory effect of PM on the production of *NO* induced by LPS stimulation. As shown in Figure 1B, the positive control, dexamethasone, which is known to be an anti-inflammatory drug, exerted a strong inhibitory effect on *NO* production. In addition, we discovered that PM dose-dependently repressed *NO* secretion to a statistically significant degree. Notably, PM inhibited *NO* production by more than 70% at a concentration of 500 μg/mL.

Further, we examined the inhibitory effect of PM on the production of the pro-inflammatory cytokines tumor necrosis factor (*TNF*)*-α*, interleukin (*IL*)*-6* and *IL-1β*. Cytokine expression was analyzed by ELISA and RT-PCR. PM did not inhibit *TNF-α* secretion (Figure 1C) and did not suppress the expression of *TNF-α* mRNA (Figure 1F). By contrast, PM effectively inhibited both *IL-6* production and mRNA expression in a dose-dependent fashion (Figure 1D,F). Likewise, PM strongly suppressed *IL-1β* cytokine and mRNA production at high concentrations (Figure 1E,F).

### 2.2. PM Strongly Suppresses Expression of iNOS but not COX-2 in LPS-Stimulated Macrophages and Induces HO-1 Induction

**Figure 2.**
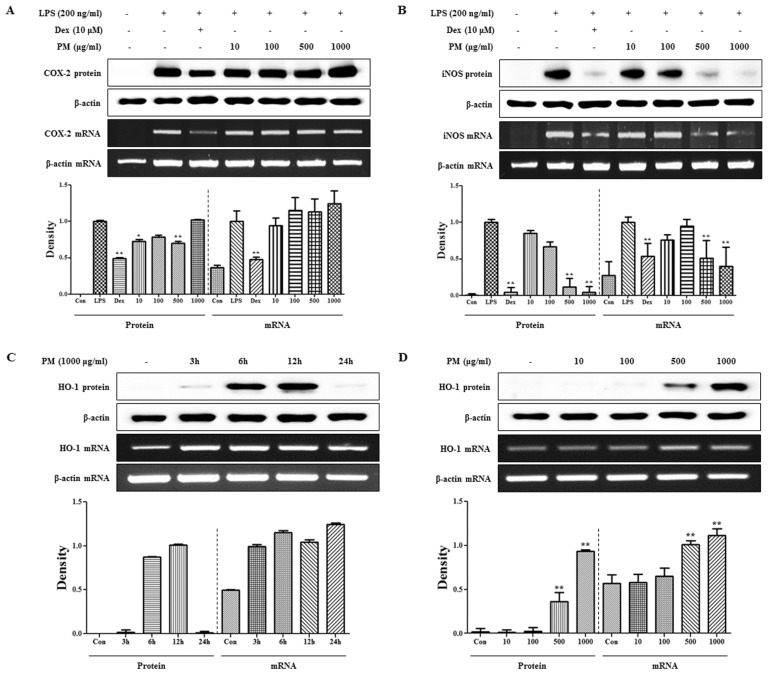
Inhibitory effect of PM on expression of (**A**) *COX-2* and (**B**) *iNOS*. And the inductive effect of PM on (**C**,**D**) *HO-1* in macrophages. Cells were treated with (**A**,**B**) LPS alone or LPS plus PM for 24 h and (**C**,**D**) with PM alone for the indicated time periods. Protein levels were determined by Western blot analysis, as described in the Materials and Methods, and quantitated using the Davinch-chemi™ CAS-400SM Chemiluminescence Imaging System (Core Bio, Seoul, Korea). Expression of mRNA was analyzed by RT-PCR. * *p* < 0.01 and ** *p* < 0.001 were calculated via comparisons with the (**A**,**B**) LPS-stimulation value or (**D**) vehicle alone.

Because *COX-2* and *iNOS* are enzymes for *PGE_2_* and *NO* synthesis, respectively, we further investigated the inhibitory effects of PM on *COX-2* and *iNOS* expression using Western blots and RT-PCR. As shown in Figure 2A, PM did not affect expression of *COX-2* at the protein or mRNA level. By contrast, PM showed a strong dose-dependent inhibitory effect on *iNOS* expression that was statistically significant (Figure 2B). The inhibitory effect of PM on *iNOS* production was believed to contribute to the suppression of *NO* secretion. These results indicate that PM has inhibitory activity against the production of pro-inflammatory mediators.

Increased *HO-1* induction has a direct effect on *iNOS* expression [8]. Therefore, we investigated whether the inhibitory effect of PM on *iNOS* expression was associated with increased *HO-1* production. We assessed *HO-1* induction in PM-treated macrophages using Western blot and RT-PCR analyses. First, we measured the induction of *HO-1* at 3–24 h after treatment with 1000 μg/mL PM. Protein and mRNA levels of *HO-1* were highest at 6 and 3 h, respectively (Figure 2C). Based on the results in Figure 2C, we investigated *HO-1* protein and mRNA expression at the indicated time points. PM induced *HO-1* expression at the protein and mRNA levels at concentrations of 500 and 1000 μg/mL in a dose-dependent manner (Figure 2D). These results suggest that pretreatment with PM inhibits *NO* and *iNOS* production by increasing *HO-1* induction.

### 2.3. PM Inhibited NF-κB Pathway Activation via Blockade of IκBα Degradation in Macrophages upon LPS Stimulation

**Figure 3.**
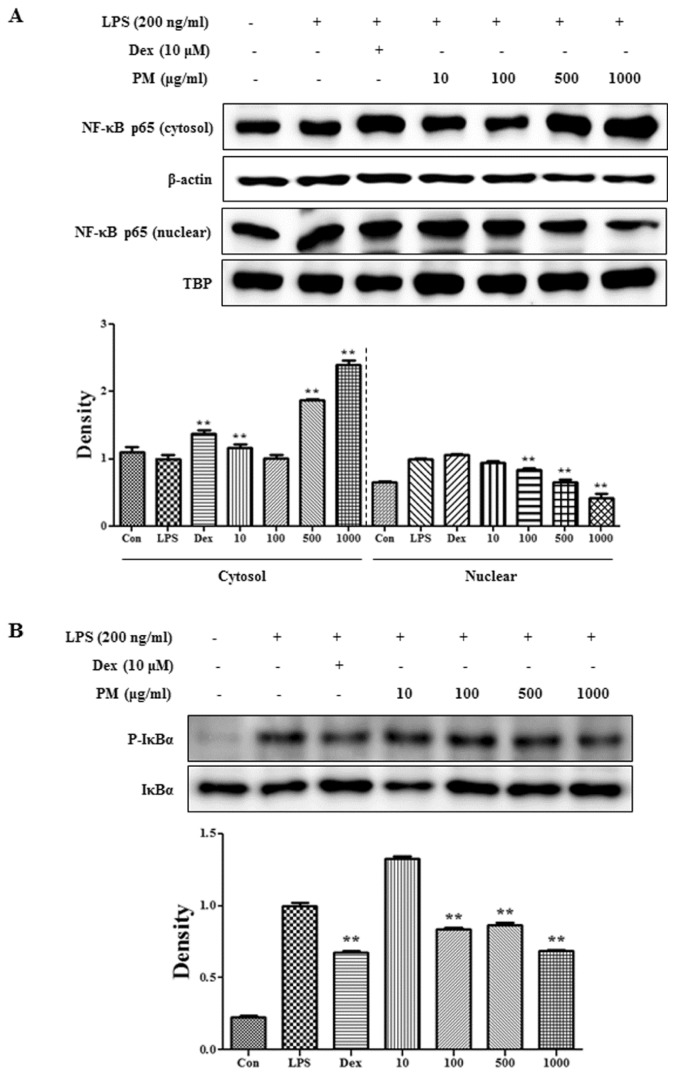
Inhibitory effects of PM on (**A**) translocation of *NF-κB* to the nucleus and (**B**) phosphorylation of *IκBα*. Cells were treated with LPS alone or with LPS and PM for 30 min (*IκBα*) or 1 h (*NF-κB*). Proteins in the cytosol or nucleus were analyzed by Western blotting. **p < 0.001 were calculated via comparisons with the LPS-stimulation value.

We demonstrated a repressive effect of PM on secretion of the inflammatory cytokine *IL-6. NF-κB* is a key transcriptional factor associated with the cellular response to stimuli, such as LPS [15–17] and with the production of *NO*, *PGE_2_*, inflammatory cytokines, and *iNOS* [18–20]. To investigate whether the inhibitory effect of PM on the expression of inflammatory mediators is associated with activity of the *NF-κB* pathway, we measured the effect of PM on *NF-κB* activation by analyzing translocation of p65 to the nucleus and the phosphorylation of *IκBα*. Western blot analysis showed that PM significantly repressed translocation of p65 to the nucleus at a concentration of 100 μg/mL or greater (Figure 3A). In addition, the phosphorylation level of *IκBα* was depressed dose-dependently after PM treatment (Figure 3B). Thus, PM inhibited the nuclear transcription of p65 by dose-dependently inhibiting *IκBα* degradation induced by LPS stimulation. These findings are consistent with previous studies showing that an *NF-κB* response drives the expression of the *iNOS* and *IL-6* genes [21–23].

### 2.4. PM Suppressed LPS-Induced Phosphorylation of MAPKs in RAW 264.7 Cells

**Figure 4.**
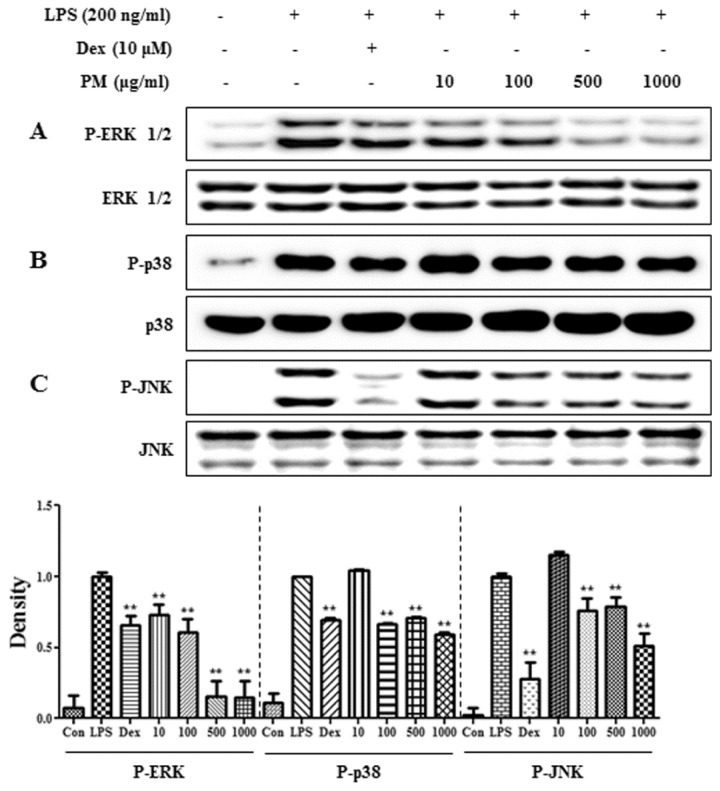
Inhibitory effect of PM on the phosphorylation of (**A**) *ERK*; (**B**) *p38* and (**C**) *JNK MAPK* in macrophages. RAW 264.7 cells were treated with PM for 30 min and then incubated with LPS for 30 min. Cell lysates were analyzed by Western blotting using specific antibodies. ** *p* < 0.001 were calculated via comparisons with the LPS-stimulation value.

Because *MAPK*s activated by phosphorylation upon LPS stimulation are related to *iNOS* expression and *NF-κB* pathway activation in macrophages [24], we examined the inhibitory effect of PM on the phosphorylation of *MAPK*s. We assessed the phosphorylation levels of *MAPK*s, including *ERK 1/2*, *p38* and *JNK*. When RAW 264.7 cells were stimulated with LPS after pretreatment with PM, the levels of phosphorylated *ERK* and *JNK MAPK* were significantly decreased with no change in non-phosphorylated *MAPK* levels (Figure 4A,C). By contrast, PM showed only a slight inhibitory effect on *p38* phosphorylation (Figure 4B). These results indicate that the inhibitory effect of PM on the phosphorylation of *MAPK*s is directly related to inhibition of *NF-κB* activation and reduced production of inflammatory factors in RAW 264.7 cells.

### 2.5. HPLC Analysis and Previous Reports on the Main Constituents of PM

**Figure 5.**
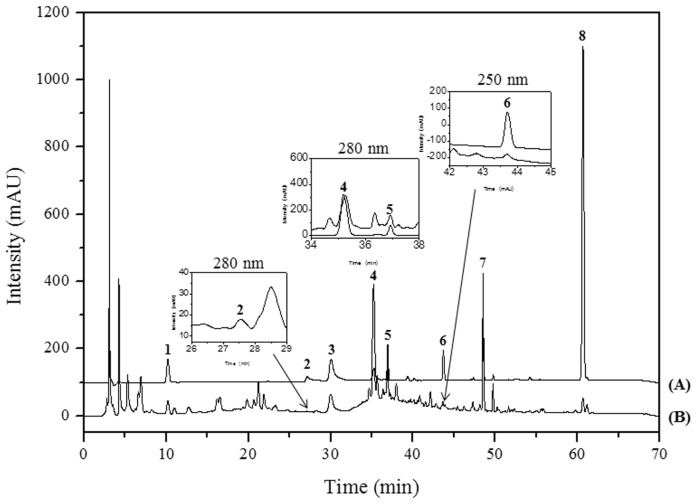
HPLC chromatograms of (A) a standard mixture and (B) PM at 250 nm. 1, 5-HMF, 10.30 min; 2, paeoniflorin, 27.22 min; 3, albiflorin, 30.28 min; 4, ferulic acid, 35.20 min; 5, nodakenin, 36.76 min; 6, decursinol, 43.86 min; 7, glycyrrhizin, 48.69 min; and 8, decursin, 60.93 min.

**Figure 6.**
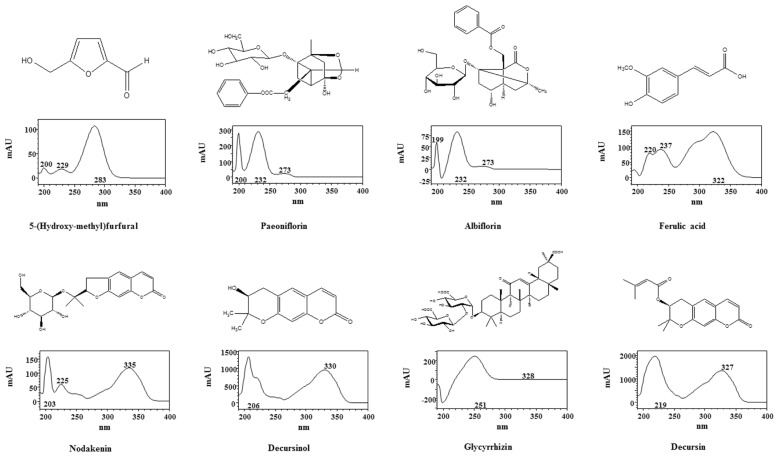
Chemical structures and HPLC DAD spectra of the main constituents of PM.

HPLC-diode array detector (DAD) analysis conditions were successfully established for the separation of peaks in PM extracts. The retention times of eight peaks were as follows: 5-hydroxymethylfurfural (5-HMF), 10.30 min; paeoniflorin, 27.22 min; albiflorin, 30.28 min; ferulic acid, 35.20 min; nodakenin, 36.76 min; decursinol, 43.86 min; glycyrrhizin, 48.69 min; and decursin, 60.93 min. Figure 5 shows chromatograms of the reference components and of a 60% methanol extract of PM, with detection of eluents at 205 nm (for decursinol), 250 nm (for 5-HMF, albiflorin, ferulic acid, nodakenin, glycyrrhizin, and decursin), 330 nm (for paeoniflorin), with ultraviolet rays (UV) wavelengths selected according to the results of Figure 6. These compounds were identified by comparing the retention time and DAD spectra with those of authentic standard compounds. Peak purity checking and identification were conducted using a 190–400 nm UV scan with a DAD.

Calibration curves were obtained using standard solutions containing 1.25–10,000 μg/mL for 5-HMF, ferulic acid, nodakenin, decursinol, glycyrrhizin, and decursin, 20–20,000 μg/mL for peaoniflorin and albiflorin as marker components. Calibration curve showed good linearity (*r*^2^ > 0.9990). The limits of detection (LOD) and limits of quantification (LOQ) were 0.16–0.50 μg/mL for 5-HMF, 0.22–0.68 μg/mL for ferulic acid, 0.13–0.40 μg/mL for nodakenin, 0.10–0.29 μg/mL for decursinol, 0.63–0.19 μg/mL for glycyrrhizin and 0.45–0.12 μg/mL for decursin, 48.17–16.05 μg/mL for peaoniflorin, 3.30–10.00 μg/mL for albiflorin (Table 1). The amounts of compounds 1–8 (5-HMF, peaoniflorin, albiflorin, ferulic acid, nodakenin, decursinol, glycyrrhizin, and decursin, respectively) were 11.09, 2.59, 2.30, 3.36, 8.23, 5.11, 0.36, and 1.17 mg/g, respectively. The analytical results for each component identified are summarized in Table 2.

A previous study reported that 5-HMF prevents *TNF-α*-induced monocytic cell adhesion to human umbilical vein endothelial cells (HUVECs) by suppression of vascular cell adhesion molecule-1 expression, reactive oxygen species generation and *NF-κB* activation [25]. Additionally, it was demonstrated that paeoniflorin suppresses *TNF-α*-induced chemokine production in human dermal microvascular endothelial cells by blocking *NF-κB* and *ERK* pathways [26]. Another recent study demonstrated that nodakenin exerts a suppressive effect on LPS-induced inflammatory responses in macrophages by inhibiting TNF receptor-associated factor 6 and *NF-κB* pathways, and it protects mice from lethal endotoxin shock [27]. A further recent study showed that glycyrrhizin inhibits *NO* and *PGE_2_* production in a bimodal fashion [28]. Another study demonstrated that decursin inhibits induction of inflammatory mediators by blocking *NF-κB* activation in macrophages [29]. These facts suggest that the anti-inflammatory activity of PM might be related to active components of PM, including 5-HMF, paeoniflorin, nodakenin, glycyrrhizin, and decursin.

## 3. Experimental Section

### 3.1. Materials and Reagents

Products related to cell culture (RPMI 1640, fetal bovine serum (FBS) and antibiotics) were purchased from Lonza (Basel, Switzerland). LPS and bovine serum albumin (BSA) were obtained from Sigma (St. Louis, MO, USA). The Cell-Counting Kit (CCK) was obtained from Dojindo Molecular Technologies, Inc. (Kumamoto, Japan). Various primary and secondary antibodies for Western blot analysis were purchased from Cell Signaling Technology, Inc. (Boston, MA, USA). Enzyme-linked immunosorbent assay (ELISA) antibody sets for cytokine detection were obtained from eBioscience (San Diego, CA, USA). An RNA extraction kit was purchased from iNtRON (Sungnam, Korea). DNA synthesizing kits and oligonucleotide primers were obtained from Bioneer (Daejeon, Korea). 5-(Hydroxy-methyl)furfural (5-HMF) and ferulic acid were purchased from Sigma (St. Louis, MO, USA). Paeoniflorin and glycyrrhizin were purchased from Tokyo Chemical Industry Co., Ltd. (Tokyo, Japan). Decursinol was purchased from Elcom Science (Seoul, Korea), nodakenin from Chem Faces (Wuhan, China), albiflorin from Wako (Osaka, Japan), and decursin from the Ministry of Food and Drug Safety (Osong, Korea). The purity of all representative standards was confirmed by high-performance liquid chromatography (HPLC) to be higher than 97%. HPLC grade solutions, acetonitrile and trifluoroacetic acid were purchased from J. T. Baker Inc. (Philipsburg, NJ, USA). Distilled water (DW) was filtered through a 0.45 μm membrane filter from ADVANTEC (Tokyo, Japan) before analysis.

### 3.2. Preparation of PM Extract

PM is composed of eight medicinal herbs listed in Table 3. All herbs were purchased from Yeongcheon Herbal Market (Yeongcheon, Korea). All voucher specimens were deposited in an herbal tank, placed in 19,200 mL of DW and then extracted by heating for 3 h at 115 °C and under high pressure (Gyeongseo Extractor Cosmos-600, Inchon, Korea). After extraction, the solution was filtered using standard testing sieves (150 μm) (Retsch, Haan, Germany), freeze-dried and kept in desiccators at 4 °C before use. The acquisition was 591 g and the yield was 30.8%. The freeze-dried extract powder was then dissolved in DW, centrifuged at 14,000 rpm for 10 min and supernatant was filtered (pore size, 0.2 μm) and kept at 4 °C prior to use.

### 3.3. Cell Culture and Drug Treatment

RAW 264.7 cells were obtained from the Korea Cell Line Bank (Seoul, Korea) and grown in complete RPMI 1640 medium. The cells were incubated in a humidified 5% CO_2_ atmosphere at 37 °C. To stimulate the cells, the medium was replaced with fresh RPMI 1640 medium, and LPS (200 ng/mL) was added in the presence or absence of various concentrations of PM (10, 100, 500, and 1000 μg/mL) for the indicated time periods.

### 3.4. Cell Viability Assay

PM was added to the cells, which were incubated for 24 h at 37 °C in 5% CO_2_. CCK solutions were added to each well, and the cells were incubated for an additional 1 h. The optical density was then read at 450 nm using an ELISA reader (Infinite M200, Tecan, Männedorf, Switzerland).

### 3.5. Determination of NO, TNF-α, IL-6 and IL-1β Cytokine Production

The cells were pretreated with PM and stimulated with LPS for 24 h. *NO* production was analyzed by measuring nitrite using Griess reagent (1% sulfanilamide, 0.1% naphthylethylenediamine dihydrochloride, 2.5% phosphoric acid) according to a previous study [30]. Secretion of the inflammatory cytokines *TNF-α*, *IL-6* and *IL-1β* was analyzed using a mouse ELISA antibody set (eBioscience, San Diego, CA, USA). The inhibitory effects of PM were determined at 570 and 450 nm for *NO* and cytokines, respectively, using an ELISA reader.

### 3.6. Preparation of Whole-Cell, Cytosolic and Nuclear Fractions and Western Blot Analysis

The expression of various proteins was analyzed by Western blot analysis according to standard procedures. Cells were stimulated with LPS with or without PM for the indicated time periods at 37 °C. After incubation, the cells were harvested and resuspended in radio immunoprecipitation assay (RIPA) lysis buffer (Millipore, Bedford, MA, USA) containing protease and phosphatase inhibitor cocktail (Roche, Basel, Switzerland) to obtain whole-cell lysates. Cytosolic and nuclear fractions were isolated using NE-PER Nuclear and Cytoplasmic Extraction Reagents (Thermo Scientific, Rockford, IL, USA) according to the procedure described by the manufacturer. After cell debris was removed by centrifugation, the concentration of protein was determined by Bradford’s method, and equal amounts of protein were separated by sodium dodecyl sulfate-polyacrylamide gel electrophoresis (SDS-PAGE). The proteins were transferred onto a nitrocellulose membrane (Millipore, Bedford, MA, USA) and blocked with 3% BSA in Tris-buffered saline containing 0.1% Tween 20 (TBS-T). The membrane was then incubated with each primary antibody at 4 °C overnight, followed by incubation with HRP-conjugated secondary antibodies. The specific proteins were detected using SuperSignal West Femto Chemiluminescent Substrate (Thermo Scientific, Rockford, IL, USA).

### 3.7. RNA Extraction and Reverse Transcription-Polymerase Chain Reaction (RT-PCR)

Total RNA was isolated using an easy-BLUE™ RNA extraction kit (iNtRON, Daejeon, Korea) according to the procedure described by the manufacturer. cDNA was synthesized using AccuPower^®^ CycleScript RT PreMix (Bioneer, Daejeon, Korea). The sequences of specific primers used for amplification by polymerase chain reaction are shown in Table 4. The following PCR conditions were applied for *TNF-α*, *IL-6*, *IL-1β*, *COX-2*, *iNOS*, *HO-1*, and β-actin: 35 cycles of denaturation at 94 °C for 30 s, annealing at the temperature indicated in Table 4 for 30 s, and extension at 72 °C for 30 s [30–34].

### 3.8. Preparation of Standard Solutions and Samples

An aqua 60% methanol standard stock solution containing compounds 5-HMF, ferulic acid, nodakenin, glycyrrhizin, decursinol, peaoniflorin, albiflorin, and decursin (each 1 mg/mL) were prepared and stored below −4 °C. Working standard solutions were prepared by serial dilution of stock solution with aqua 60% methanol. All calibration curves were obtains from assessement of peak areas of standards in the concentration ranges. A sample of 10 mg PM extract was prepared in 1 mL DW, extracted by ultra-sonication, and filtered through a 0.2 μm syringe membrane filter from Whatman Ltd. (Maidstone, UK) before injection into the HPLC system for analysis. Sample solutions were stored at −4 °C in a refrigerator before analysis.

### 3.9. General Experimental Procedures

Analytical HPLC data were obtained using an L-2130 pump, L-2200 auto-sampler, L-2300 column oven and L-2455 UV/VIS DAD. The output signal of the detector was recorded using EZChrom Elite software for the HPLC system (Hitachi, Tokyo, Japan). The OptimaPak C_18_ analytical HPLC column (4.6 × 250 mm, 5 μm; RS Tech Co., Daejeon, Korea) was used in this study.

### 3.10. Analytical Chromatographic Conditions

The mobile phase consisted of water containing (A) 0.1% trifluoroacetic acid and (B) acetonitrile with gradient elution at a flow rate of 1.0 mL/min. The sample injection volume was 20 μL, and the flow rate of the mobile phase was 1.0 mL/min (Table 5). The column temperature was maintained at 40 °C, and the wavelengths of the UV detector were set at 205, 250, and 330 nm.

### 3.11. Statistical Analysis

The results are expressed as mean ± SE values. Statistical significance for each treated group compared with the negative control was determined using the Student’s *t* test. Each experiment was repeated at least three times to yield comparable results. *p* values of <0.01 and <0.001 were considered significant.

## 4. Conclusions

In conclusion, PM shows significant inhibitory effects on the secretion of *NO* and expression of *IL-6*, *IL-1β* and *iNOS* in LPS-stimulated RAW 264.7 cells. These effects are due to inhibition of *NF-κB* activation through suppression of *IκBα* degradation and blockade of *MAPK* phosphorylation. Furthermore, the induction of *HO-1* by PM inhibits inflammatory factor production. These results show that PM could be developed as a new anti-inflammatory agent derived from natural products.

## Figures and Tables

**Table 1.** Linearity, correlation coefficient, limits of detection (LOD), and limits of quantification (LOQ) of the marker compound (*n* = 3).

Compound	Linear Range (μg/mL)	Regression Equation a	Correlation Coefficient (*r*^2^)	LOD b (μg/mL)	LOQ c (μg/mL)
5-HMF	1.25–10,000	*y* = 403917*x* − 87212	0.9993	0.16	0.50
Ferulic acid	1.25–10,000	*y* = 273782*x* + 89791	0.9998	0.23	0.68
Nodakenin	1.25–10,000	*y* = 239585*x* − 25958	1.0000	0.13	0.40
Decursinol	1.25–10,000	*y* = 598246*x* + 235041	0.9996	0.10	0.29
Glycyrrhizin	1.25–10,000	*y* = 43887*x* + 38994	0.9991	0.63	0.19
Decursin	1.25–20,000	*y* = 116410*x* + 1188529	1.0000	0.45	0.12
Peaoniflorin	20–20,000	*y* = 1871.8*x* + 16715	0.9990	16.05	48.17
Albiflorin	20–20,000	*y* = 8026.8*x* + 6805.9	0.9993	3.33	10.00

a *y* = peak area (mAU) of the components, *x* = concentration (μg·mL^−1^) of the components;

b LOD = 3× signal-to-noise (*S*/*S*) ratio;

c LOQ = 10× signal-to-noise (*S*/*S*) ratio.

**Table 2.** Content of the eight marker compounds of Palmultang (*n* = 3).

Compound	Content (mg/g)		

	Mean	SD	RSD (%)
5-HMF	11.09	0.35	3.11
Ferulic acid	2.59	0.00	0.15
Nodakenin	2.30	0.00	0.02
Decursinol	3.36	0.02	0.72
Glycyrrhizin	8.23	0.01	0.13
Decursin	5.11	0.00	0.01
Peaoniflorin	0.36	0.00	0.34
Albiflorin	1.17	0.03	2.41

**Table 3.** Herbal components and amount of Palmultang (PM) decoction.

Herbs	Amount of Herbs (g)
Ginseng Radix	240
Atractylodes Rhizome White	240
Poria	240
Glycyrrhizae Radix et Rhizoma	240
Angelica Gigas Root	240
Prepared Rehmannia Root	240
Peony Root	240
Cinidium Rhizome	240
Total weight	1920

**Table 4.** Primer sequences and annealing temperatures used for RT-PCR analysis.

Target Gene	Primer Sequence	Annealing Temp
*TNF-α*	F: 5′-AGCACAGAAAGCATGATCCG-3′ R: 5′-GTTTGCTACGACGTGGGCTA-3′	55 °C
*IL-6*	F: 5′-CATGTTCTCTGGGAAATCGTGG-3′ R: 5′-AACGCACTAGGTTTGCCGAGTA-3′	58 °C
*IL-1β*	F: 5′-TGCAGAGTTCCCCAACTGGTACATC-3′ R: 5′-GTGCTGCCTAATGTCCCCTTGAATC-3′	64 °C
*COX-2*	F: 5′-CACTCAGTTTGTTGAGTCATTC-3′ R: 5′-GATTAGTACTGTAGGGTTAATG-3′	45 °C
*iNOS*	F: 5′-AGCCCAACAATACAAATGACCCTA-3′ R: 5′-TTCCTGTTGTTTCTATTTCCTTTGT-3′	56 °C
*HO-1*	F: 5′-TGAAGGAGGCCACCAAGGAGG-3′ R: 5′-AGAGGTCACCCAGGTAGCGGG-3′	62 °C
*β-actin*	F: 5′-ATGAAGATCCTGACCGAGCGT-3′ R: 5′-AACGCAGCTCAGTAACAGTCCG-3′	58 °C

F, forward; R, reverse.

**Table 5.** HPLC conditions used for the analysis of PM.

Item	Condition		
Mobile phase	Time (min)	Water (Containing 0.1% TFA)	Acetonitrile

	0	5	95
	5	5	95
	15	15	85
	25	15	85
	50	65	35
	60	65	35

Flow rate	1.0 mL/min		

Inject volume	20 μL		

Column	OptimaPak C_18_ (4.6 × 250 mm, 5 μm, RS tech Co., Daejeon, Korea)		

Column temperature	40 °C		

UV wavelength	205, 250 and 330 nm		
